# Association of alcohol consumption with sleep disturbance among adolescents in China: a cross-sectional analysis

**DOI:** 10.3389/fpubh.2025.1564292

**Published:** 2025-06-16

**Authors:** Yanhong Liu, Lei Zhang, Meng Yao, Yanrou Li, Kang Cao, Yongzheng Deng

**Affiliations:** Shenzhen Bao’an Center for Chronic Disease Control, Shenzhen, Guangdong, China

**Keywords:** alcohol, sleep quality, adolescent, PSQI, cross-sectional analysis

## Abstract

**Background:**

Evidence suggests a potential association between alcohol consumption and sleep quality. However, knowledge of this association among adolescents in China is limited. Thus, this study aims to investigate the association between alcohol consumption and sleep quality in a group of adolescents in China.

**Methods:**

A cross-sectional survey was conducted in Shenzhen, Guangdong Province in China from October 2021 to December 2021. Alcohol consumption refers to drinking more than half a bottle/a can of beer, a small cup of white wine/foreign wine and a glass of wine/rice wine/fruit wine/highland wine at a time in the past year. Sleep quality was measured using the Pittsburgh Sleep Quality Index (PSQI) with a cutoff >5 indicating sleep disturbance. Multivariable logistic regression models were utilized to estimate the association.

**Results:**

A total of 2,505 adolescents were included in the analysis. Among them, the mean age was 14.36 years (SD 1.74); 58.08% were male, and 41.92% were female. The overall drinking rate was 26.07%, and the median score of PSQI was 5. The interquartile distance of the PSQI global score was 4. Multivariable logistic regression illustrated that alcohol consumption exhibited positive significant associations with poor PSQI scores (OR = 1.89, 95% CI: 1.52–2.35) compared with good scores.

**Conclusion:**

Alcohol consumption is associated with poor sleep quality in adolescents in China accounting for socioeconomic contexts and psychosocial stressors. The findings underscore the public health urgency of addressing alcohol drinking behaviors to mitigate sleep disturbances in adolescents. Further studies need to be performed to explore the causality between alcohol consumption and sleep quality in adolescents. Frequency and dosage of alcohol consumption need to be considered to explore dose–response relationships.

## 1 Introduction

Quality sleep is important for the growth and development of adolescents, as well as for keeping their physical and psychological health (1). This type of sleep is characterized by sufficient duration, good quality and the absence of daytime sleepiness (2). Sleep problems have been shown to negatively impact the mental health of adolescents, increasing the risk of several mental health issues, including anxiety, depression, suicidal behavior, and attention disorders (3–5).

Mental health problems can also lead to sleep disorders. Sleep disorders nearly inevitably occur in people with depression (6, 7) According to the cognitive model of insomnia, excessive and uncontrollable anxiety, fear and intrusive thoughts reduce sleep quality and exacerbate insomnia (8, 9). Studies have shown a two-way link between sleep problems and anxiety symptoms in adolescents (10, 11). The National Sleep Foundation recommends that adolescents in the US sleep between 8 and 10 h per night (12), but recent research has estimated that adolescents reported obtaining 7 h of nighttime sleep on average.

Several studies (13, 14) have found that roughly 70% of adolescents get less than 8 h of sleep per day, with 16.7% of teens (15) rating their sleep quality as poor. Research shows the percentage of adolescents in Brazil, the United Kingdom, Japan, and China who self-report insufficient sleep duration or poor sleep quality ranges from 25.7 to 45.9% (16–20).

Numerous detrimental lifestyle habits can affect sleep health. A significant negative association has been observed between alcohol use and sleep quality (21). Acute and chronic alcohol consumption can cause sleep disturbances that may persist even after abstinence in alcoholics (22). In adults, alcohol decreases sleep onset latency and sleep efficiency and increases wakefulness after sleep onset (23). In addition, sleep in alcoholics during acute drinking is characterized by prolonged sleep latency and decreased total sleep time (24). The adolescent brain develops unevenly, with the regions responsible for processing emotions and impulses maturing earlier than those governing rational decision-making. This imbalance leads adolescents to act more impulsively and engage in risk-taking behaviors such as alcohol drinking when confronted with uncertain situations (25). The adolescent population exhibits a higher prevalence rate of sleep-alcohol comorbidity. Several studies in United States and Japan have shown that there is a significant association between sleep problems in adolescents and alcohol use. The symptoms of adolescent sleep disorders such as initial insomnia, difficulty falling asleep, subsequent shortened sleep duration and daytime sleepiness are positively correlated with the frequency and amount of alcohol use (26–28).

Although limited study (29, 30) have explored alcohol use and sleep in Chinese adolescents, none comprehensively examined this relationship considering socioeconomic and psychosocial factors. Our analysis extended the scope by systematically incorporating socioeconomic determinants and psychosocial factors, thereby providing a more robust estimation of the independent association between alcohol use and sleep quality.

## 2 Methods

### 2.1 Study design and participant

Cross-sectional data analysis was performed using data from a national survey on alcohol-related behaviors among adolescents, which was sponsored and supervised by the National Health Commission of China. This study conducted a cross-sectional school survey on middle and high school adolescents in Bao’an District of Shenzhen City from October 2021 to December 2021 and targeted adolescents aged 12–17 years.

The sample size was calculated based on an estimated prevalence of sleep problems of 35%, with an *α* of 0.05 and *δ* of 0.02, which resulted in a sample size of 2,185. We expanded our sample size by 15% to 2,512 to address potential sampling errors and the possibility of refusals or invalid questionnaires. A total of 2,688 adolescents were enrolled from 8 junior high schools, 5 ordinary high schools and 2 vocational high schools by multi-stage stratified cluster random sampling. Multi-stage stratified cluster random sampling involved: (1) stratifying schools by type (junior high, ordinary high, vocational high); (2) randomly selecting 15 schools (8 junior, 5 ordinary, 2 vocational) using a random number generator; (3) within each school, randomly selecting 4–6 classes (cluster size: 30–50 adolescents) to participate, with all adolescents in selected classes included. Adolescents who failed to complete questionnaires were excluded. As a result, 2,505 participants remained for the analysis.

The Ethical Review Committee of the Bao’an Center for Chronic Disease Control, Shenzhen, reviewed and approved the study. All adolescents and their parents or guardians were fully informed that participation was completely voluntary, and informed consent was obtained in writing from the parents or guardians of all participants for this survey. All research methods were conducted in accordance with relevant guidelines and regulations. Trained researchers implemented the survey in classrooms and instructed the adolescents on how to complete the survey. All questionnaires were completed anonymously and collected on-site in classrooms to ensure confidentiality. To ensure that adolescents provided accurate and truthful information, the researchers thoroughly explained the purpose of the study to the participants. They emphasized that the study was unrelated to academic performance, there were no right or wrong answers, and that all data collected would remain strictly confidential and used solely for scientific research purposes. This approach aimed to create a safe and non-judgmental environment for participants to share their experiences honestly.

### 2.2 Outcome assessment

Sleep quality was evaluated using the Pittsburgh Sleep Quality Index (PSQI, Chinese version), which has been frequently used to assess sleep quality (23, 24) It is constructed with self-reported questions measuring sleep quality and disturbance over a time interval of 1 month. It contains 19 items in 7 components, including subjective sleep quality, sleep latency, sleep duration, sleep efficiency, sleep disturbance, use of sleeping medication and daytime dysfunction. The score range of each component is 0 to 3, and the global score ranges from 0 to 21. A higher PSQI score indicates poorer sleep quality and vice versa (31). The PSQI has been widely used in most previous studies and exhibited high sensitivity (82–83%) and specificity (77–85%) (32, 33). We classified all adolescents into two groups according to their sleep quality global score of 5 as the cutoff point. A global score > 5 was recognized as poor sleep quality, and a global score ≤ 5 was regarded as good sleep quality (34).

### 2.3 Alcohol consumption assessment

(1) Alcohol consumption was defined as drinking more than half a bottle/can of beer, a small cup of white wine, or equivalent alcohol content (≥10 g) on at least one occasion in the past year. Occasional attempts do not count.” Occasional attempts” were defined as isolated instances not meeting the threshold (≥10 g ethanol per occasion). (2) Drinking rate refers to the proportion of adolescents who have drunk alcohol at any time in the past year in the total number of adolescents surveyed.

### 2.4 Covariates

Sociodemographic: General demographic characteristics, including age, gender, weight, height, accommodation, school type, father’s educational level, mother’s educational level and pocket money, were collected using a standard questionnaire. Body mass index (BMI) was calculated as body weight divided by squared height (kg/m^2^).

Depression: The Center for Epidemiological Studies Depression Scale (CES-D, Chinese version), which was originally developed in English by Radloff in 1977 (35), and its Chinese version was later cross-culturally adapted through standardized translation procedures conducted by Zhang et al., with Cronbach’s *α* = 0.90 (36). It contains 20 items and has been widely used in epidemiological surveys to screen for symptoms of depression In this study, a low score of 15 or below indicated no depressive symptoms,16 to19 indicated likely depressive symptoms, and 20 or above indicated definite depressive symptoms (37).

Perceived stress: Perceived stress was measured using the Chinese version of the Perceived Stress-14 (PSS-14) (38), which was originally developed by Cohen et al. in 1983 (39). The Chinese version of the Perceived Stress-14 (PSS-14) has good validity, with Cronbach’s *α* = 0.85 (40). It contains 14 items and is a widely used instrument for measuring the perception of stress. In this study, a low score of 0 to 14 indicated low stress levels, 15 to 28 indicated moderate stress levels, 29 to 42 indicated high stress levels and 43 to 56 indicated extremely high stress levels (41).

Social support: Perceived social support was measured using the Chinese version of the Perceived Social Support Scale (PSSS) (42), which was originally developed by Zimet et al. (43). The scale consists of 12 items assessing support from family, friends, and others, with Cronbach’s *α* = 0.88 (44). In this study, a low score of 12 to 36 indicated low perceived social support levels, 37 to 60 indicated moderate perceived social support levels and 61 to 84 indicated high perceived social support levels (45).

### 2.5 Statistical analysis

Means ± standard deviations were used to describe the concentrated and discrete trends of continuous variables. Numbers and percentages were utilized to describe categorical variables. For the description of sleep, we used the median, interquartile range (P25, P75) and minimum and maximum values to describe the PSQI and its 7 component scores, given that the PSQI followed a non-normal distribution. A chi-square test was conducted for categorical variables.

We used a binary logistic regression model to evaluate the association between alcohol consumption and the prevalence of poor sleep quality. We first developed an unadjusted model to assess the relationship between alcohol consumption and sleep quality. Then, we developed an adjusted model controlling for age, gender, BMI, accommodation, school type, pocket money, depression, perceived stress and social support. The results from the adjusted model were considered the main findings. Depression (CES-D), perceived stress (PSS-14), and social support (PSSS) scores differed significantly between sleep quality groups (all *p* < 0.001), supporting their inclusion as covariates in the multivariable model.

All statistical analyses were performed using SPSS20.0, and a two-tailed *p* < 0.05 was applied to assess the statistical significance.

## 3 Results

### 3.1 Drinking rate and demographic characteristics

First, a total of 2,688 adolescents were enrolled in the study. Among them, 183 participants were eventually excluded owing to illogical or incomplete responses to the questionnaire, with a completion rate of 93.19%. Last, 2,505 participants were enrolled in the analysis. Out of the 2,505 participants, 1,003 had a total PSQI score greater than 5, accounting for 40.04% of the total. The participants had a mean age of 14.36 years (SD 1.74). Among the 2,505 participants, 58.08% were male, and 41.92% were female. The overall drinking rate in adolescents was 26.07%. Except for the father’s educational level and the mother’s educational level, other variables were significantly different across the groups of PSQI scores. These variables were included in the multivariable logistic regression model. Details are shown in Table 1.

**Table 1:** Analysis of participants’ demographic status, depression, perceived stress and social support in relation to PSQI.

Characteristic	Total (*N* = 2,505)	PSQI scores		*p* value
		≤5 (*N* = 1,502)	>5 (*N* = 1,003)	
Age (years), mean ± SD	14.36 ± 1.74	14.03 ± 1.68	14.86 ± 1.70	<0.001
Sex, n (%)				0.028
Male	1,455(58.08)	899(59.85)	556(55.43)	
Female	1,050(41.92)	603(40.15)	447(44.57)	
School type, n (%)				<0.001
Middle school	1,589(63.43)	1,073(71.44)	516(51.45)	
High school	604(24.11)	256(17.04)	348(34.70)	
Vocational high school	312(12.46)	173(11.52)	139(13.86)	
BMI, mean ± SD	19.98 ± 4.07	19.65 ± 4.03	20.48 ± 4.07	<0.001
Accommodation, n (%)				<0.001
Boarding at school	1,101 (43.95)	570(37.95)	531(52.94)	
Staying at home	1,404 (56.05)	932(62.05)	472(47.06)	
Father’s educational level, n (%)				0.379
Primary school and below	323(12.89)	196(13.05)	127(12.66)	
Middle school	628(25.07)	360(23.97)	268(26.72)	
High school	710(28.34)	430(28.63)	280(27.92)	
College	379(15.13)	241(16.05)	138(13.76)	
Undergraduate and above	465(18.56)	275(18.31)	190(18.94)	
Mother’s educational level, n (%)				0.818
Primary school and below	431(17.21)	253(16.84)	178(17.75)	
Middle school	658(26.27)	385(25.63)	273(27.22)	
High school	691(27.58)	420(27.96)	271(27.02)	
College	353(14.09)	216(14.38)	137(13.66)	
Undergraduate and above	372(14.85)	228(15.18)	144(14.36)	
Pocket money, n (%)				<0.001
none	854(34.09)	548(36.48)	306(30.51)	
1–35 yuan/week	512(20.44)	340(22.64)	172(17.15)	
36–70 yuan/week	327(13.05)	192(12.78)	135(13.46)	
71–140 yuan/week	313(12.50)	175(11.65)	138(13.76)	
141–210 yuan/week	254(10.14)	137(9.12)	117(11.67)	
211and above yuan/week	245(9.78)	110(7.32)	135(13.46)	
Alcohol consumption, n (%)				<0.001
No	1852(73.93)	1,235(82.22)	617(61.52)	
Yes	653(26.07)	267(17.78)	386(38.48)	
CES-D				<0.001
≤15	1,594(63.63)	1,213(80.76)	381(37.99)	
16–19	271(10.82)	119(7.92)	152(15.15)	
≥20	640(25.55)	170(11.32)	470(46.86)	
PSS-14				<0.001
0–14	429(17.13)	366(24.37)	63(6.28)	
15–28	1,340(53.49)	894(59.52)	446(44.47)	
29–42	674(26.91)	237(15.78)	437(43.57)	
43–56	62(2.47)	5(0.33)	57(5.68)	
PSSS				<0.001
12–36	145(5.79)	31(2.06)	114(11.37)	
37–60	1,052(42.00)	536(35.69)	516(51.45)	
61–84	1,308(52.21)	935(62.25)	373(37.18)	

### 3.2 Description of PSQI

The detailed conditions of sleep quality in participants are illustrated in Table 2. Among all adolescents, the median score of PSQI was 5. The interquartile distance of the PSQI global score was 4. The minimum value of the PSQI global score was 0, and the maximum value was 19. Among the seven specific components of the PSQI global score, sleep quality, sleep latency, sleep disturbance and sleep dysfunction had a median of 1. In the meantime, the three other components had a median of 0. Details are shown in Table 2.

**Table 2:** Description of sleep quality.

Domains	Med	IQD	P_25_	P_75_	Minimum	Maximum
PSQI global score	5	4	3	7	0	19
(C1) Sleep quality	1	0	1	1	0	3
(C2) Sleep latency	1	2	0	2	0	3
(C3) Sleep duration	0	1	0	1	0	3
(C4) Sleep efficiency	0	0	0	0	0	3
(C5) Sleep disturbance	1	1	0	1	0	3
(C6) Sleep medication	0	0	0	0	0	3
(C7) Sleep dysfunction	1	1	1	2	0	3

### 3.3 Association of sleep quality with alcohol consumption

Logistic regression was conducted to assess the association between alcohol consumption with sleep quality. From the results of the adjusted model, alcohol consumption showed positive significant associations with a poor PSQI score (OR = 1.89, 95% CI: 1.52–2.35) compared with a good one. Moreover, alcohol consumption was positively associated with scores on each of the specific seven dimensions of the PSQI. The results of the unadjusted model are consistent with those of the adjusted model. Details are shown in Table 3.

**Table 3:** Logistic regression analyses of alcohol assumption and sleep quality.

Domains	Alcohol assumption		Unadjusted model		Adjusted model	
	No (*N* = 1,852)	Yes (*N* = 653)	OR (95% CI)	*p* value	OR (95%CI)	*p* value
PSQI level						
Good (≤5)	1,235	267	Ref	*/*	Ref	*/*
Poor (>5)	617	386	2.89(2.41, 3.48)	<0.001	1.89 (1.52, 2.35)	<0.001
(C1) Sleep quality, n (%)						
0	514	100	Ref		Ref	*/*
≥1	1,338	553	2.12(1.68,2.69)	<0.001	1.36(1.05, 1.77)	*0.022*
(C2) Sleep latency, n (%)						
0	861	200	Ref	*/*	Ref	*/*
≥1	991	453	1.97 (1.63,2.38)	<0.001	1.46(1.19, 1.81)	<0.001
(C3) Sleep duration, n(%)						
0	1,105	268	Ref	*/*	Ref	*/*
≥1	747	385	2.13 (1.77, 2.55)	<0.001	1.40(1.13,1.72)	0.002
(C4) Sleep efficiency, n(%)						
0	1,610	519	Ref	*/*	Ref	*/*
≥1	242	134	1.72 (1.36, 2.17)	<0.001	1.42(1.10, 1.84)	0.007
(C5) Sleep disturbance, n (%)						
0	550	101	Ref	*/*	Ref	*/*
≥1	1,302	552	2.31 (1.83, 2.92)	<0.001	1.74(1.34, 2.24)	<0.001
(C6) Sleep medication, n (%)						
0	1827	621	Ref	*/*	Ref	*/*
≥1	25	32	3.77 (2.21, 6.40)	<0.001	2.44(1.36, 4.38)	0.003
(C7) Sleep dysfunction, n (%)						
0	517	75	Ref	*/*	Ref	*/*
≥1	1,335	578	2.99 (2.30, 3.88)	<0.001	1.96(1.47, 2.62)	<0.001

Adjusted covariates including age, gender, BMI, accommodation, school type, pocket money, depression, perceived stress and social support. The Nagelkerke pseudo R^2^ for the final model was 0.31, indicating that 31% of the variability in sleep quality was explained by the predictors.

## 4 Discussion

Sleep disturbance is common in adolescence. This study found that 40% of adolescents in Shenzhen, China, do not have good sleep quality, which is similar to the situation of adolescents in Brazil and Turkey. In Brazilian adolescents, the prevalence of short sleep duration and the negative perception of sleep quality increased in 10 years from 31.2% in 2001 to 45.9% in 2011 (46). Bedir found that 41.1% of adolescents had poor sleep quality in the Gebze District of Kocaeli in Turkey (47). A study found that two-thirds of adolescents reported insufficient sleep in the US (5). Omotoso found that three out of every five adolescents were poor sleepers (PSQI global score > 5) with a cross-sectional design among 512 in-school adolescents in north central Nigeria (48). Sleep duration and sleep quality seriously affect the physical and mental health of adolescents and can cause many health problems, including poor academic performance, poor eating habits, cardiometabolic risk, obesity and insulin resistance (49). This study found that teenagers’ mental health differed significantly between sleep quality groups, which is consistent with several other studies (50, 51).

Alcohol consumption among adolescents is also a serious problem in countries around the world. In the United States more than 60% of 12th-grade adolescents reported having used alcohol in their lifetime, and nearly 35% reported using alcohol in the past month (52). In Spain, a survey showed that, among 2,865 adolescents, 1,681 reported drinking alcohol at some point in their lives; 1,208 said they had drunk alcohol in the previous year, accounting for 42.2% of the total sample (53). Our study found that the current drinking rate among adolescents is 26.07% in Shenzhen, China, which is lower compared with that of United States and Spain.

The hazards of alcohol consumption level include long-term health risks and short-term risks related to acute poisoning, including impairment of cognitive and psychomotor performance and cardiovascular function, sleep interruption and cardiac vagal tension during sleep, and impairment of emotional and cognitive function (54–57).

Some literature has studied the effect of drinking alcohol on sleep quality. The association of alcohol dependence with insomnia is likely to be bidirectional in nature (55). A significant, negative within-person association was observed between sleep quality and alcohol use. Sleep quality was lower on nights following alcohol use (21). Research shows that alcohol interferes with sleep through various mechanisms, such as interfering with the electrophysiological sleep structure, causing insomnia, initiating abnormal circadian rhythm, shortening sleep duration, increasing sleep latency, decreasing sleep volume (21, 58, 59), reducing sleep quality (60, 61). Notably, about 50% of alcohol-dependent patients suffer from insomnia (62).

Alcohol can also increase respiratory-related sleep events, such as snoring and decreased oxygen saturation (63). In addition, alcohol can aggravate other sleep disorders, such as sleep apnea and periodic limb movements. Many of these altered sleep patterns even persist during the long-term recovery of alcohol consumption (64).

The reasons why drinking alcohol affects sleep quality are complex. Alcohol is a small water-soluble molecule that rapidly distributes throughout the body and has a wide range of effects. It has a negative impact on many organ systems and destroys almost all neurobiological mechanisms in various ways. Therefore, it is difficult to determine the specific mechanism by which ethanol affects sleep and its associated diseases. Studies have found that at low doses, alcohol mainly affects the function of the central nervous system by interfering with the normal functioning of neurotransmitters gamma aminobutyric acid and glutamate, which also play a crucial role in wakefulness sleep states (56). In addition, some studies have found that alcohol can lead to the accumulation of adenosine. The interaction between the adenosine mechanism and the anorexigenic system in the hypothalamic fornix area is key to the impact of alcohol on sleep (65).

Our study advances prior research on alcohol use and sleep quality in Chinese adolescents by adopting a multidimensional analytical framework that integrates both socioeconomic and psychosocial factors, thereby providing a more robust estimation of the independent association between alcohol use and sleep quality. However, this study also has several limitations that should be acknowledged. First, the cross-sectional design precludes causal inference between alcohol consumption and sleep quality. Second, reliance on self-reported PSQI scores may introduce subjective bias, as sleep quality was not objectively validated through polysomnography (PSG), the gold standard for diagnosing sleep disorders (66). Lastly, we could not conduct dose–response analysis (e.g., frequency or quantity of alcohol use) due to data restrictions.

## 5 Conclusion

Alcohol consumption was significantly associated with poor sleep quality among Chinese adolescents, even after adjusting for socioeconomic factors and psychosocial variables. The findings underscore the public health urgency of addressing alcohol drinking behaviors to mitigate sleep disturbances in adolescents. Further studies need to be performed to explore the causality between alcohol consumption and sleep quality in adolescents. Frequency and dosage of alcohol consumption need to be considered to explore dose–response relationships.

## Data Availability

The datasets presented in this article are not readily available because the datasets used and/or analyzed during the current study are available from the corresponding author upon reasonable request. Requests to access the datasets should be directed to Lei Zhang, zlsuc1981@163.com.

## References

[1] Kaur H Bhoday HS. Changing adolescent sleep patterns: factors affecting them and the related problems. J Adolesc Health. (2017) 65:73–7. doi: 10.1016/j.jadohealth.2017.06.023 28462547

[2] Buysse DJ. Sleep health: can we define it? Does it matter? Sleep. (2014) 37:9–17. doi: 10.5665/sleep.3298, PMID: 24470692 PMC3902880

[3] Gregory AM Sadeh A. Sleep, emotional and behavioral difficulties in children and adolescents. Sleep Med Rev. (2012) 16:129–36. doi: 10.1016/j.smrv.2011.03.007, PMID: 21676633

[4] Kaneita Y Ohida T Osaki Y Tanihata T Minowa M Suzuki K. Association between mental health status and sleep status among adolescents in Japan: a nationwide cross-sectional survey. J Clin Psychiatry. (2007) 68:1426–35. doi: 10.4088/jcp.v68n0916, PMID: 17915984

[5] Pereira T Martins S Fernandes L. Sleep duration and suicidal behavior: a systematic review. Eur Psychiatry. (2017) 41:s854. doi: 10.1016/j.eurpsy.2017.01.1699

[6] Yasugaki S Okamura H Kaneko A Hayashi Y. Bidirectional relationship between sleep and depression. Neurosci Res. (2025) 211:57–64. doi: 10.1016/j.neures.2023.04.006, PMID: 37116584

[7] Riemann D Krone LB Wulff K Nissen C. Sleep, insomnia, and depression. Neuropsychopharmacology. (2020) 45:74–89. doi: 10.1038/s41386-019-0411-y, PMID: 31071719 PMC6879516

[8] Narmandakh A Roest AM Jonge P Oldehinkel AJ. The bidirectional association between sleep problems and anxiety symptoms in adolescents: a TRAILS report. Sleep Med. (2020) 67:39–46. doi: 10.1016/j.sleep.2019.10.018, PMID: 31887607

[9] Peltz JS Rogge RD Pugach CP Strang K. Bidirectional associations between sleep and anxiety symptoms in emerging adults in a residential college setting. Sage Publications. (2017) 5:204–15. doi: 10.1177/2167696816674551

[10] Kelly RJ El-Sheikh M. Reciprocal relations between children’s sleep and their adjustment over time. Dev Psychol. (2014) 50:1137–47. doi: 10.1037/a0034501, PMID: 24188035

[11] McMakin DL Alfano CA. Sleep and anxiety in late childhood and early adolescence. Curr Opin Psychiatry. (2015) 28:483–9. doi: 10.1097/YCO.0000000000000204, PMID: 26382163 PMC4670558

[12] McKnight-Eily LR Eaton DK Lowry R Croft JB Presley-Cantrell L Perry GS. Relationships between hours of sleep and health-risk behaviors in US adolescent adolescents. Prev Med. (2011) 53:271–3. doi: 10.1016/j.ypmed.2011.06.020 21843548

[13] Loessl B Valerius G Kopasz M Hornyak M Riemann D Voderholzer U. Are adolescents chronically sleep-deprived? An investigation of sleep habits of adolescents in the southwest of Germany. Child Care Health Dev. (2008) 34:549–56. doi: 10.1111/j.1365-2214.2008.00845.x, PMID: 18549435

[14] Hoefelmann LP Silva KS Barbosa Filho VC Silva JA Nahas MV. Behaviors associated to sleep among high school students: cross-sectional and prospective analysis. Rev Bras Cineantropom Desempenho Hum. (2014) 16:68–78. doi: 10.5007/1980-0037.2014v16n1p68

[15] Brooks F Magnusson J Klemera E Spencer N Smeeton N. HBSC England national report: Health behaviour in school-aged children. Hatfield: University of Hertfordshire (2015).

[16] Ohida T Osaki Y Doi Y Tanihata T Minowa M Suzuki K. An epidemiologic study of self-reported sleep problems among Japanese adolescents. Sleep. (2004) 27:978–85. doi: 10.1093/sleep/27.5.978, PMID: 15453558

[17] Xu Z Su H Zou Y Chen J Wu J Chang W. Sleep quality of Chinese adolescents: distribution and its associated factors. J Paediatr Child Health. (2012) 48:138–45. doi: 10.1111/j.1440-1754.2011.02065.x, PMID: 21470332

[18] Zhou Y Guo L Lu C Deng J He Y Huang J. Bullying as a risk for poor sleep quality among high school adolescents in China. PLoS One. (2015) 10:e0121602. doi: 10.1371/journal.pone.0121602 25811479 PMC4374746

[19] Lydon DM Ram N Conroy DE Pincus AL Geier CF Maggs JL. The within-person association between alcohol use and sleep duration and quality in situ: an experience sampling study. Addict Behav. (2016) 61:68–73. doi: 10.1016/j.addbeh.2016.05.018, PMID: 27249804 PMC4915974

[20] Department for Transport. Contributory factor, reported accidents by severity. London: Department for Transport (2016).

[21] Chan JK Trinder J Andrewes HE Colrain IM Nicholas CL. The acute effects of alcohol on sleep architecture in late adolescence. Alcohol Clin Exp Res. (2013) 37:1720–8. doi: 10.1111/acer.12141, PMID: 23800287 PMC3987855

[22] Brower KJ. Alcohol’s effects on sleep in alcoholics. Alcohol Res Health. (2001) 25:110–25. 11584550 PMC2778757

[23] Xie Y Xiang H Di N Mao Z Hou J Liu X. Association between residential greenness and sleep quality in Chinese rural population. Environ Int. (2020) 145:106100. doi: 10.1016/j.envint.2020.106100, PMID: 32916416

[24] Wang Y Li Y Liu X Liu R Mao Z Tu R. Gender-specific prevalence of poor sleep quality and related factors in a Chinese rural population:the Henan rural cohort study. Sleep Med. (2019) 54:134–41. doi: 10.1016/j.sleep.2018.10.031, PMID: 30554057

[25] Willoughby T Good M Adachi PJ Hamza C Tavernier R. Examining the link between adolescent brain development and risk taking from a social-developmental perspective. Brain Cogn. (2013) 83:315–23. doi: 10.1016/j.bandc.2013.09.008, PMID: 24128659

[26] Marmorstein NR. Sleep patterns and problems among early adolescents: associations with alcohol use. Addict Behav. (2017) 66:13–6. doi: 10.1016/j.addbeh.2016.11.002, PMID: 27863322

[27] Morioka H Itani O Kaneita Y Ikeda M Kondo S Yamamoto R. Associations between sleep disturbance and alcohol drinking: a large-scale epidemiological study of adolescents in Japan. Alcohol. (2013) 47:619–28. doi: 10.1016/j.alcohol.2013.09.041, PMID: 24188738

[28] Haynie DL Lewin D Luk JW Lipsky LM O’Brien F Iannotti RJ. Beyond sleep duration: bidirectional associations among chronotype, social jetlag, and drinking behaviors in a longitudinal sample of US high school students. Sleep. (2018) 41:zsx202. doi: 10.1093/sleep/zsx202, PMID: 29237053 PMC6018914

[29] Chen H Bo QG Jia CX Liu X. Sleep problems in relation to smoking and alcohol use in Chinese adolescents. J Nerv Ment Dis. (2017) 205:353–60. doi: 10.1097/NMD.0000000000000661, PMID: 28181924

[30] Su H Lyu D Huang K Yan J. Association of physical activity, screen time, and sleep with substance use in children and adolescents: a large-sample cross-sectional study. Front Public Health. (2024) 12:1432710. doi: 10.3389/fpubh.2024.1432710, PMID: 39484350 PMC11524877

[31] Buysse DJ Reynolds CF Monk TH Berman SR Kupfer DJ. The Pittsburgh sleep quality index: a new instrument for psychiatric practice and research. Psychiatry Res. (1989) 28:193–213. 2748771 10.1016/0165-1781(89)90047-4

[32] Tang J Liao Y Kelly B Xie L Xiang Y Qi C. Gender and regional differences in sleep quality and insomnia: a general population-based study in Hunan Province of China. Sci Rep. (2017) 7:43690. doi: 10.1038/srep43690 28262807 PMC5337959

[33] Tsai P Wang S Wang M Su C Yang T Huang C. Psychometric evaluation of the Chinese version of the Pittsburgh sleep quality index (CPSQI) in primary insomnia and control subjects. Qual Life Res. (2005) 14:1943–52. doi: 10.1007/s11136-005-4346-x, PMID: 16155782

[34] Çelebioğlu A Aytekin Özdemir A Küçükoğlu S Ayran G. The effect of internet addiction on sleep quality in adolescents. J Child Adolesc Psychiatr Nurs. (2020) 33:221–8. doi: 10.1111/jcap.12287, PMID: 32657485

[35] Radloff LS. The CES-D scale: a self-report depression scale for research in the general population. Appl Psychol Meas. (1977) 1:385–401.

[36] Zhang J Wu ZY Fang G Li J Han BX Chen ZY. Establishment of national urban norm of Center for Epidemiological Studies Depression Scale. Chin Ment Health J. (2010) 24:139–41. doi: 10.3969/j.issn.1000-6729.2010.02.014

[37] Ying J Yap P Gandhi M Liew TM. Validity and utility of the Center for Epidemiological Studies Depression Scale for detecting depression in family caregivers of persons with dementia. Dement Geriatr Cogn Disord. (2019) 47:323–34. doi: 10.1159/000500940, PMID: 31307034 PMC6878745

[38] Yang TZ Huang HT. An epidemiological study on stress among urban residents in social transition period. Chin J Epidemiol. (2003) 24:760–3. 14521764

[39] Cohen S Kamarck T Mermelstein R. A global measure of perceived stress. J Health Soc Behav. (1983) 24:385–95. 6668417

[40] Leung DY Lam TH Chan SS. Three versions of perceived stress scale: validation in a sample of Chinese cardiac patients who smoke. BMC Public Health. (2010) 10:513. doi: 10.1186/1471-2458-10-513, PMID: 20735860 PMC2939644

[41] Shah M Hasan S Malik S Sreeramareddy CT. Perceived stress, sources and severity of stress among medical undergraduates in a Pakistani medical school. BMC Med Educ. (2010) 10:2. doi: 10.1186/1472-6920-10-2, PMID: 20078853 PMC2820489

[42] Jiang QJ. Perceived social support scale. Chin J Behav Med Brain Sci. (2001) 10:41–2.

[43] Zimet GD Dahlem NW Zimet SG Farley GK. The multidimensional scale of perceived social support. J Pers Assess. (1988) 52:30–41. 10.1080/00223891.1990.9674095 2280326

[44] Wang Y Gu J Zhang F Xu X. The mediating role of social support and resilience between self-efficacy and prenatal stress: a mediational analysis. BMC Pregnancy Childbirth. (2023) 23:866. doi: 10.1186/s12884-023-06184-2, PMID: 38104088 PMC10724952

[45] Dong S Ge H Su W Guan W Li X Liu Y. Enhancing psychological well-being in college adolescents: the mediating role of perceived social support and resilience in coping styles. BMC Psychol. (2024) 12:393. doi: 10.1186/s40359-024-01902-7 39010140 PMC11250932

[46] World Health Organization. Programming for adolescent health and development. World Health Organ Tech Rep Ser. (1999) 886:1–260. 10352574

[47] Bedir Y Gündoğdu F Şişman FN Ergün A. Relationship between sleep quality and emotion-behavior problems in adolescents. J Turk Sleep Med. (2020) 7:17–23. doi: 10.4274/jtsm.galenos.2020.46220

[48] Omotoso AB Abdulmalik JO Adediran KI Omigbodun OO. Sleep quality and its correlates among adolescents schooling in northcentral Nigeria. Res J Health Sci. (2022) 10:216–23. doi: 10.4314/rejhs.v10i3.6

[49] Otsuka Y Kaneita Y Itani O Jike M Osaki Y Higuchi S. The relationship between subjective happiness and sleep problems in Japanese adolescents. Sleep Med. (2020) 69:120–6. doi: 10.1016/j.sleep.2020.01.008, PMID: 32062038

[50] Kouros CD Keller PS Martín-Piñón O El-Sheikh M. Bidirectional associations between nightly sleep and daily happiness and negative mood in adolescents. Child Dev. (2022) 93:e547–62. doi: 10.1111/cdev.13798, PMID: 35596680 PMC9545079

[51] Bacaro V Miletic K Crocetti E. A meta-analysis of longitudinal studies on the interplay between sleep, mental health, and positive well-being in adolescents. Int J Clin Health Psychol. (2023) 24:100424. doi: 10.1016/j.ijchp.2023.100424 38125984 PMC10730350

[52] Bagot KS. Editorial: durability of alcohol use prevention effects in adolescents and transitional age youth. J Am Acad Child Adolesc Psychiatry. (2022) 61:473–5. doi: 10.1016/j.jaac.2021.12.001, PMID: 34921908

[53] Prieto-Ursúa M Baena B. Alcohol consumption in adolescents: the predictive role of drinking motives. Psicothema. (2020) 32:189–96. doi: 10.7334/psicothema2019.263 32249744

[54] Pabon E Greenlund IM Carter JR de Wit H. Effects of alcohol on sleep and nocturnal heart rate: relationships to intoxication and morning-after effects. Alcohol Clin Exp Res. (2022) 46:1875–87. doi: 10.1111/acer.14921, PMID: 35953878 PMC9826048

[55] Colrain IM Crowley KE Nicholas CL Padilla M Baker FC. The impact of alcoholism on sleep evoked delta frequency responses. Biol Psychiatry. (2009) 66:177–84. doi: 10.1016/j.biopsych.2008.10.010, PMID: 19058790 PMC3987847

[56] Roehrs T Roth T. Sleep, sleepiness, and alcohol use. Alcohol Res Health. (2001) 25:101–9. 11584549 PMC6707127

[57] Van Reen E Tarokh L Rupp TL Seifer R Carskadon MA. Does timing of alcohol administration affect sleep? Sleep. (2011) 34:195–205. doi: 10.1093/sleep/34.2.195, PMID: 21286495 PMC3022940

[58] Alam MN McGinty D. Acute effects of alcohol on sleep are mediated by components of homeostatic sleep regulatory system. J Neurochem. (2017) 142:620–3. doi: 10.1111/jnc.14100, PMID: 28736837

[59] Ebrahim IO Shapiro CM Williams AJ Fenwick PB. Alcohol and sleep I: effects on normal sleep. Alcohol Clin Exp Res. (2013) 37:539–49. doi: 10.1111/acer.12006, PMID: 23347102

[60] Colrain IM Nicholas CL Baker FC. Alcohol and the sleeping brain. Handb Clin Neurol. (2014) 125:415–31. doi: 10.1016/B978-0-444-62619-6.00024-0, PMID: 25307588 PMC5821259

[61] Britton A Fat LN Neligan A. The association between alcohol consumption and sleep disorders among older people in the general population. Sci Rep. (2020) 10:5275. doi: 10.1038/s41598-020-62227-0, PMID: 32210292 PMC7093458

[62] Wiers CE. Adenosine sheds light on the relationship between alcohol and sleep. J Neurosci. (2014) 34:7733–4. doi: 10.1523/JNEUROSCI.1274-14.2014, PMID: 24899696 PMC6608262

[63] He S Hasler BP Chakravorty S. Alcohol and sleep-related problems. Curr Opin Psychol. (2019) 30:117–22. doi: 10.1016/j.copsyc.2019.03.007, PMID: 31128400 PMC6801009

[64] Mukherjee S Kazerooni M Simasko SM. Dose-response study of chronic alcohol induced changes in sleep patterns in rats. Brain Res. (2008) 1208:120–7. doi: 10.1016/j.brainres.2008.02.079, PMID: 18387599 PMC2435013

[65] Sharma R Sahota P Thakkar MM. Orexin, alcohol and sleep homeostasis. Orexin and Sleep. Springer International Publishing. (2015):137–64. doi: 10.1007/978-3-319-23078-8_9

[66] Malhotra RK Kirsch DB Kristo DA Olson EJ Aurora RN Carden KA. Polysomnography for obstructive sleep apnea should include arousal-based scoring: an American Academy of sleep medicine position statement. J Clin Sleep Med. (2018) 14:1245–7. doi: 10.5664/jcsm.7234, PMID: 29991439 PMC6040795

